# Ought Asylums to Rank as Hospitals?

**Published:** 1886-10-16

**Authors:** 


### OUGHT ASYLUMS TO RANK AS HOSPITALS?

The Asylums will Co-operate.

WE very cordially welcome the expressions of sympathy and interest which asylum superintendents and their officers have already shown in THE HOSPITAL. One of our objects was to emphasise the fact that asylums, rightly administered and understood, must be regarded as hospitals. It is not generally known, or at any rate not generally understood, that, owing to the large number of chronic invalids who seek relief at the hospitals, a new departure has been made at Birmingham, and also in the United States, where chronic hospitals have been erected and opened. These chronic hospitals will receive and provide for no patients but those whose ailments are of such long standing as to preclude their admission into an ordinary hospital which is intended for the treatment of acute cases of illness. In the same way there are of course acute and chronic cases of insanity, and in recently constructed asylums this fact has been recognised by the provision of hospital blocks in addition to the ordinary blocks and those for special cases. Whether it will be possible, or even desirable, to attempt to extend the system we are considering, by dividing asylums for the insane into acute and chronic asylums, as hospitals are now being divided, we are not yet prepared to say.

Asylums as Hospitals.

Dr. Savage, we believe, has striven to make Bethlehem an acute asylum, and few, if any, chronic cases are now admitted there. It would be very interesting to have his experience, and to compare it with that of Mr. John Jaffray of Birmingham, the founder of chronic hospitals. Meanwhile, whether the asylums of the future are to be divided into two great groups, like the general hospitals, or whether each asylum, as we think will prove to be best, continues to provide special accommodation for both classes of patients, no reasonable person can doubt that the asylums as at present conducted are entitled to rank as hospitals, a fact which must prove of inestimable advantage to the public and also to the medical profession. It is our mission to widen the area of interest in all hospitals, and for this reason the sympathy and co-operation of asylum superintendents and their officers will, we hope, be cordially extended to this Journal.

We claim to be Hospital Men.

Dr. A. R. Urquhart, of James Murray's Royal Asylum, Perth, writes:—"We, the asylum superintendents and our officers, constantly claim to be hospital men, and the large and important work which engages us should secure your good-fellowship in such undertakings. The addition of the names of our leading representatives would strengthen your hands, and most of us could say something of value in regard to the subject-matter in your opening address." We are much indebted to Dr. Urquhart, and we cordially thank him for this expression of opinion. It will prove to the outside world that asylums are not now conducted without hope, or brightness, or faith; but, on the contrary, that love and a desire to keep touch with the outer world, find a large place in the systems on which these institutions are, for the most part, now conducted.

Will the Asylums help?

Next week we hope to publish an article on the training of asylum attendants, male and female. Dr. Urquhart and others may be glad to know that we are anxious to co-operate with the asylum superintendents; that we shall at all times welcome items of news, notices of appointments, events, and changes of staff, and contributions for the asylum column, where we propose to print, at Dr. Hack Tuke's suggestion, the autobiography of patients in regard to their malady. Besides Dr. Hack Tuke, Dr. Richard Greene of the Northampton County Asylum, Dr. Wallis of Whittingham, Dr. Cowles of the Maclean Asylum, Somerville, Mass., and one of the leading authorities in Scotland, whose name we are not at liberty to mention, are all willing to help to develop this branch of the hospital field. Any others who may be interested, and who will co-operate in the endeavour to make THE HOSPITAL of real use and interest to asylum superintendents and their officials, will be heartily welcomed. Meanwhile we hope that there will soon be few, if any, people who will doubt that every well-conducted asylum is entitled to rank as a hospital.

